# Notes and News

**Published:** 1886-12-11

**Authors:** 


### NOTES AND NEWS.

COLLECTED AND COMMUNICATED.

THE London Hospital, in the death of Mr. O. E. Coope, has lost one of the staunchest supporters this institution ever possessed. The deceased gentleman was a Life Governor of the London all his life. In 1873 he gave a sum of £5,000 towards the special fund for the erection of the new wing and the maintenance of the hospital. At the last meeting of the Governors, a vote of condolence was passed to Mrs. Coope, when Mr. Carr Gomme referred to the sad loss which the institution had sustained.

THERE is nothing which hospital managers value more than annual subscriptions. The Secretary of St. Mary's Hospital, Mr. Michelli, sends us the following letter which he received on the 2nd inst. "In answer to the appeal of the Marquis of Salisbury, the Misses —— forward £—— as a subscription to St. Mary's Hospital." One indirect result of the Hospitals Week was that one large general hospital in London, which issued a thousand appeals during the weeks immediately preceding and following it, received in response no less than £1,000 in varying amounts. It would be interesting to know how many of the hospitals throughout England have received contributions since Lord Salisbury's appeal appeared in these columns. We shall be glad to have any in formation and facts bearing upon this subject.

A DRAMATIC performance was given on Saturday evening at the Novelty Theatre, in aid of the Maternity Fund of St. Mary's Hospital at Paddington. *New Men and Old Acres* was played before a large house, and the institution is likely to be a considerable gainer.

THE year of Jubilee is fast approaching, and all orders of men who are loyal to their country and their constitution, are looking forward to its celebration on a scale proportionate to its importance. From the days of the Norman Conquest to our own, but three monarchs have enjoyed a reign of fifty years. The fourth jubilee is now at hand, and it is earnestly to be hoped that the friends of the Hospital Sunday Fund will make the collections of 1887 so unprecedently large, that they will go far to fill the pinched exchequers of our charities. This year the Fund produced many thousand pounds over and above the collection of last year, thanks to the enthusiasm created by the meetings held during the Hospital Week. Next year, is it too much to expect that the citizens of London will mark the year of jubilee by a thank-offering of £80,000?

THE good people of Bournemouth have now finally determined that the form which their memorial of the Queen's Jubilee shall take shall be a hospital for the sick poor of the town. The name, the Victoria Hospital, was easily and readily decided upon, but the site is still under consideration. Doctors say that West Bournemouth is more bracing than east. As there are also some admirable sites available, it is to be hoped the hospital will be erected to the westward.

We are glad to learn, from a notice given by Sir Edmund H. Currie at the last meeting of the Council of the Hospital Sunday Fund, that a special effort will be made in the Royal Jubilee Year to relieve the hospitals and medical charities of the Metropolis from the "unprecedentedly terrible straits" (to use Sir Edmund's words) "in which they are now placed."

---

The Board of Delegates of the Hospital Saturday Fund resolved at their meeting on Saturday to distribute the sum of £9,750 for the present year. Lord Brassey was elected President, in the place of the late Mr. Morley.

---

The Bristol Eye Hospital, the third oldest Ophthalmic institution in this country, is, after a career of seventy-five years' useful work in very humble premises, about to be considerably enlarged and improved. Two adjacent houses are to be purchased at a cost of £1,300. Already the extension fund amounts to £2,000, towards the £3,500 required.

---

By a majority of one only, the Liverpool Hospital Sunday Committee have decided to include the Shaw Street Hospital for Women in the list of charities receiving help from the fund. This narrow vote should serve to remind the managers of the hospital in question that public opinion is not yet at ease with regard to the recent scandals which have arisen.

---

Despite the prevailing depression which exists, it is pleasing to notice that here and there a charity is able to pursue the even tenor of its way. The Royal Hospital for Incurables at Putney is in this fortunate position. The income of this institution during the past year has not fallen off, and the usual election of fifty to the benefits of this excellent institution has accordingly been made. In that fine edifice on Putney Heath there are now 192 inmates, while the pensioners number 481, making a grand total of 673 *bénéficiaires*. During the past twelve months the Putney Hospital for Incurables has received over £8,000 in the shape of legacies.

---

A Hospital for Infectious Diseases is about to be erected at Tolworth, near Long Ditton, for the Rural Sanitary Authority of Kingston-on-Thames. The architects are Messrs. Jacomb, Gibbon, and Woodroffe, of Verulam Buildings, W.C., and the amount of the accepted tender is £7,850. The tenders for the work varied in amount from over £12,000 to £6,000.

---

On Thursday, in last week, the 16th annual course of winter entertainments for the in-patients of the National Hospital for the Paralysed and Epileptic was commenced. The programme, which was very kindly provided by Miss Moran, contained an excellent selection of music, vocal and instrumental, and appeared to give thorough satisfaction to the patients and their friends. The next entertainment, to be provided by Mrs. Gibson, will take place on the 16th inst.

---

The following vacancies are announced this week, the amount of the salary being placed in each case after the name of the institution:—

**Resident Medical Officers.**

Norfolk County Lunatic Asylum. Medical Superintendent.—£450, and furnished house.
South Devon Hospital, Plymouth. House Surgeon.—£100, with board.
Royal Hospital for Diseases of the Chest, City Road. Junior House Physician.—£50, with board.
East London Hospital for Children. Clinical Assistant.—Board; no salary.
Manchester Royal Infirmary. Doubly qualified.—£150, with board.

**Non-Resident Medical Officers.**

Bristol Dispensary. Doubly qualified.—One vacancy.

**Matrons.**

Western General Dispensary, Marylebone.—£60, and rooms.
Middlesbrough Fever Hospital.—£60, with board.

**Trained Nurses.**

Royal Hospital for Diseases of the Chest, City Road.—£22, with uniform.
Chester Diocesan Institution. Two.—£24, with uniform.
Bradford Infirmary.—£25, with uniform.
Kent and Canterbury Institute. One. Medical and Surgical.
Thrapston Union.—£25, with board.

---

The Committee of Management of the Colnbrook Orphan Asylum offer a prize of three hundred guineas for the first, and one hundred guineas for the second best design, for their new Orphanage. Particulars are to be had of Mr. Nevil Sinclair, 18, Princess Street, Westminster, on payment of 10*s*.

---

Miss E. Littlewood, who was trained at the London School of Medicine and Royal Free Hospital, is now on her passage out to Bombay, to take up her appointment as Resident Medical Officer to the Cama Hospital, an institution which was opened last summer, entirely for the treatment of the native women of India.

---

The Globe Parcel Delivery Co., 4, Wood Street, Liverpool, has offered to convey to the Mill Road Infirmary, free of charge, any parcels of illustrated papers, books, periodicals, toys, etc., which the wealthier portion of the inhabitants may be willing to send for the benefit or amusement of the sick and destitute inmates of that institution. The Company also undertake the collection of such packages in their vans, on receipt of a postcard from the donors requesting them to call. We know by experience that many people, who cannot afford or are unwilling to pay the cost of carriage, would gladly send such contributions to the hospitals and infirmaries if they could get them there without trouble or expense. What a boon, therefore, to the patients it would be if other large carrying firms throughout the kingdom would follow the kindly example of the Liverpool Globe Delivery Company. We shall be happy to publish the names of any Parcels' Delivery Co. which will do what is here suggested, and to print their arrangements and rules.

---

"Mother" writes us: "Is there any room in your columns set apart for the little ones, for any little story or relation of anything, such as a little story, entitled 'Annals of the Nursery,' or 'Angel Sister,' or such like?" We have not, as our "Children's Corner" shows, been altogether unmindful of our little readers, and we hope in the future to do still more for them. "Mother's"

suggestion is, we think, an exceedingly good one, and we shall be glad to receive from her, or any other correspondent, some contributions of the class referred to.

---

The Rev. Fairfax Goodall, chaplain of Bristol Royal Infirmary, appeals to the public through the columns of the *Bristol Western Daily Press* for gifts of suitable books for the use of patients in the convalescent ward. Here is one opening, not only for our readers, but also for the great carrying companies, to evince their goodwill and benevolence towards such of their poorer brethren as are passing through the weariness and monotony of convalescence. How weary, and how monotonous, only those who have suffered from severe illness know.

---

With the object of obtaining pecuniary aid for the Beau Site Convalescent Home at Hastings, two entertainments, consisting of vocal and instrumental music and *tableaux vivants*, were given in the Royal Concert Hall of that town on the 23rd and 24th ult. Large audiences attended, and the performances were successful in every way.

---

The Committee of the Keyham and Dockyard (Plymouth) employés interested in procuring funds for the Royal Albert Hospital, Devonport, state in their nineteenth annual report, that £91 11*s.* 5*d.* has been raised by means of halfpenny weekly subscriptions alone, during the year ending 30th September last. In the preceding year £67 10*s.* 5½*d.* was raised by the same agency; and the comparison is of interest, showing, as it does, the increased and increasing popularity of the hospitals with the classes who are directly benefited by them.

---

The Governors of Sherburn Hospital, Durham, have found it necessary to memorialise the Charity Commissioners with regard to the increased expenditure necessary to maintain the efficiency of their institution. They ask that the total deficit of £3,054 shall be made up from unappropriated funds, and that their income shall be permanently increased from £4,850 to £5,500 per annum. In consequence, Mr. Walker Skirrow held an exhaustive inquiry on the 26th ult., and has reported to the Commissioners, with what result we have yet to learn.

---

A French lady named Laborie, who died lately, has bequeathed the sum of 800,000 francs to the City of Paris, to be distributed in gifts of 25 francs each amongst the convalescents leaving the Refuge at Vincennes. We do not know what the accommodation at this Refuge may be, but a bequest which extends its benefits to 32,000 individuals in succession seems likely to last no inconsiderable time.

---

Lord Aberdare appeals, in *The Times* of November 30th, for help to reduce the large deficit of £3,100 in the working accounts of the Hospital for Sick Children in Great Ormond Street, of which he is the Chairman. Although other hospitals with a like object have been built in various parts of the metropolis, and have doubtless intercepted many contributions which would otherwise have been bestowed upon the Great Ormond Street Charity, the demands upon the latter for indoor and outdoor treatment have not been thereby diminished in the slightest degree. "A belief prevails in many quarters," writes Lord Aberdare, "that the Children's Hospital is possessed of adequate if not superabundant resources, but I much regret that this is not the case."

---

Colonel Milman, of the Governor's House, Camden Road, N., has been elected chairman of the Camden District Committee of the Great Northern Central Hospital, and has already received the following donations: Mr. James Hughes, 50 guineas; Mr. William Ashe, £10; Mr. Thomas Kell, £5; Mr. Jesse Jones, 1 guinea. If Colonel Milman can secure a similar list each week throughout the year, he will prove himself to be a Chairman of Chairmen.

---

In reference to the paragraph on the Kyrle Society in our impression of the 27th ult., Miss E. S. Busk writes to inform us that flowers sent to the Society should be consigned to Miss Tripp, who undertakes their distribution among the hospitals, etc. Miss Busk superintends the Literature Branch of the Society at 11, Nottingham Place, W., the object of which is to distribute among poor people the old books which the well-to-do cast from their libraries. Speaking of the flowers, we might, perhaps, advise intending givers to send, a week or so in advance, a letter of intimation to the hon. sec. of the Flower Branch, who will inform them which institutions, at the current moment, are most in need of the particular kind of flowers intended to be sent. By this means the friends of this Society are enabled to send the flowers direct to the institution while they are in a fresh condition.

---

Fact, we know, is often far stranger than fiction. A short time since, we narrated a remarkable incident at the London Hospital, and now one comes from the Homerton Infirmary. A young man was recently conveyed to this institution with a broken thigh-bone. His young brother came to visit him, he tripped, fell heavily, and broke *his* thigh-bone. The two brothers are at the present moment occupying adjacent beds, both suffering from like injuries. There is material enough here for the delectation of those who revel in theories of probabilities.

---

The new infirmary buildings at Workington were opened on the 29th ult., by Mrs. Alexander Wilson, of Sheffield. These contain three large wards, and have been erected at a cost of more than £2,000, exclusive of furnishing expenses. The ceremony was duly honoured by the presentation of a gold key to the lady-opener by Mr. Peill, the Chairman of the Building Committee.

---

At a recent sitting of the select committee appointed to inquire into the condition of the Melbourne Hospital, Dr. Youl (the coroner) and Dr. Girdlestone, both condemned the present building, and urged the necessity of its reconstruction or removal. Dr. Youl recommended the detached-cottage system to the favourable consideration of the committee.

YET another bazaar! Hastings has hitherto been without a hospital worthy of the name. The handsome building which has just been erected on White Rock will prove both an architectural adornment to the town, and an incalculable boon to the sick poor. The bazaar, which is in aid of the Building Fund, opens on Wednesday next, and will be continued on Thursday and Friday.

---

THE late Mr. Caledon George Du Pré, of Wilton Park, Bucks, has left £300 to the Brompton Consumption Hospital, similar amounts to King's College, and the British Orphan Asylum, Slough, and £200 to the Buckingham County Infirmary at Aylesbury.

---

REFERRING to our annotation on the subject of the Guest Hospital, Dudley, we are glad to find that the complaint about the difficulty of obtaining information made by *The Wolverhampton Chronicle* was of old date. We learn from an authoritative source that, since the appointment of the present Resident Medical Officer, Dr. Corrie Keep, every facility has been afforded to the Press for the obtaining of desired information. It is a satisfaction to us to receive and publish this disclaimer.

---

THE Cheltenham Hospital is one of those institutions which are greatly in need of help from the benevolent at the present time. Certain additions, amongst which are a new operating-room and an accident ward, together with several structural alterations, have been found necessary, and will entail a heavy call upon the invested capital, unless extraneous aid is forthcoming, as we trust may be the case.

---

THE abuses to which such forcible attention was called some time ago in connection with the Aberdeen Royal Infirmary are now in the fair way of being removed. All who have had the best opportunities of knowing, and are best qualified to judge, unite in testifying to the really good work that has been accomplished. Evidence is daily accumulating of the wisdom of the step the managers took in putting the care of the institution into the skilful and experienced hands of Miss Lumsden. But though much has already been accomplished, and the work of reform has been put on lines in which it cannot fail to be carried to a successful issue, much yet remains to be done. The great question of structural improvement has yet to be decided, and even though the prospects of a final agreement on it are becoming brighter every day, the inevitable money difficulty has still to be tackled.

---

IT was eminently gratifying to those interested in the prosperity of the Aberdeen Hospital to find the newly elected Lord Provost of the city, Mr. William Henderson, alluding in his opening address to the Town Council to the urgent character of the claims which the hospital had upon all citizens. Mr. Henderson made this very happy suggestion, that there could be no more fit memorial of the Jubilee year of our Queen's reign than a hospital thoroughly equipped according to modern requirements. This certainly argues well for the future of the infirmary.

